# Alkalosis and Dialytic Clearance of Phosphate Increases Phosphatase Activity: A Hidden Consequence of Hemodialysis

**DOI:** 10.1371/journal.pone.0159858

**Published:** 2016-07-25

**Authors:** Ricardo Villa-Bellosta, Emilio González-Parra, Jesús Egido

**Affiliations:** 1 Fundación Instituto de Investigación Sanitaria, Fundación Jiménez Díaz (FIIS-FJD). Madrid, Spain; 2 Spanish Biomedical Research Network in Diabetes and Associated Metabolic Disorders (CIBERDEM), Madrid, Spain; 3 Renal Division, “Fundación Jiménez Díaz” University Hospital, Madrid Autonomous University, Madrid, Spain; The University of Tokyo, JAPAN

## Abstract

**Background:**

Extracellular pyrophosphate is a potent endogenous inhibitor of vascular calcification, which is degraded by alkaline phosphatase (ALP) and generated by hydrolysis of ATP via ectonucleotide pyrophosphatase/phosphodiesterase 1 (eNPP1). ALP activity (as routinely measured in clinical practice) represents the maximal activity (in ideal conditions), but not the real activity (in normal or physiological conditions). For the first time, the present study investigated extracellular pyrophosphate metabolism during hemodialysis sessions (including its synthesis via eNPP1 and its degradation via ALP) in physiological conditions.

**Methods and Findings:**

45 patients in hemodialysis were studied. Physiological ALP activity represents only 4–6% of clinical activity. ALP activity increased post-hemodialysis by 2% under ideal conditions (87.4 ± 3.3 IU/L *vs*. 89.3 ± 3.6 IU/L) and 48% under physiological conditions (3.5 ± 0.2 IU/L *vs*. 5.2 ± 0.2 IU/L). Pyrophosphate synthesis by ATP hydrolysis remained unaltered post-hemodialysis. Post-hemodialysis plasma p*H* (7.45 ± 0.02) significantly increased compared with the pre-dialysis p*H* (7.26 ± 0.02). The slight variation in p*H* (~0.2 units) induced a significant increase in ALP activity (9%). Addition of phosphate in post-hemodialysis plasma significantly decreased ALP activity, although this effect was not observed with the addition of urea. Reduction in phosphate levels and increment in p*H* were significantly associated with an increase in physiological ALP activity post-hemodialysis. A decrease in plasma pyrophosphate levels (3.3 ± 0.3 μmol/L *vs*. 1.9 ± 0.1 μmol/L) and pyrophosphate/ATP ratio (1.9 ± 0.2 *vs*. 1.4 ± 0.1) post-hemodialysis was also observed.

**Conclusion:**

Extraction of uremic toxins, primarily phosphate and hydrogen ions, dramatically increases the ALP activity under physiological conditions. This hitherto unknown consequence of hemodialysis suggests a reinterpretation of the clinical value of this parameter.

## Introduction

Vascular calcification (VC), or ectopic calcification in vessels, is highly prevalent in patients with chronic hemodialysis, and is associated with cardiovascular events and all-cause mortality[1,2]. Elevated serum phosphorus and calcium phosphate products are a predictable accompaniment in chronic hemodialysis patients in the absence of dietary phosphate restrictions or supplemental phosphate binders, and contribute to the substantial morbidity and mortality rates in this population[2]. The calcium phosphate product has demonstrated a mortality risk similar to that of phosphate alone[3,4]. Although plasma phosphate and calcium phosphate product correlate with mortality risk in chronic hemodialysis patients, a number of studies indicate that this is not the full explanation. Medial calcification is commonly observed in diabetes, aging, and several genetic disorders[5–9], with normal plasma calcium and phosphate concentrations[7].

Extracellular fluids, including serum and plasma, are supersaturated with plasma phosphate and calcium levels, resulting in a tendency toward spontaneous calcium phosphate crystal (CPC) deposition, a hallmark of VC[10]. A potent endogenous physicochemical inhibitor of CPC formation and growth is extracellular pyrophosphate (PPi)[11]. In extracellular fluids, PPi is generated enzymatically by extracellular ATP hydrolysis via ectonucleotide pyrophosphatase/phosphodiesterase (eNPP) and is degraded to Pi by alkaline phosphatase (ALP)[10]. Overexpression of ALP in cells is sufficient to cause *ex vivo* medial calcification in aortic rings[12]. The addition of ALP in culture media causes vascular smooth muscle calcification[12], and *in vivo* overexpression of ALP in smooth muscle cells results in excessive VC[12]. In two murine models demonstrating excessive VC, increased expression and activity of ALP was reported[8,13]. Inhibition of ALP was observed to reduce VC in a mouse model overexpressing ALP in smooth muscle cells[12]. On the other hand, eNPP1 deficiency in humans markedly reduces plasma PPi levels and results in extensive VC, which is referred to as idiopathic infantile arterial calcification[5]. Moreover, eNPP1-null mice develop ectopic artery calcification[14], which is restored by addition of eNPP1[15].

An appropriate balance in Pi and PPi homeostasis is required to prevent CPC deposition[16]. Phosphate homeostasis has been extensively studied in patients undergoing chronic hemodialysis. Current treatment options, including dietary phosphate restriction, supplemental phosphate binders, serum alkalinization, and supplementation with calcimimetics, are aimed at reducing abnormalities in Pi homeostasis and associated acidosis[17]. Despite these efforts, VC associated with chronic hemodialysis has not improved. The main aim of this study was to analyze extracellular PPi metabolism (including its synthesis and degradation) during hemodialysis sessions to establish an efficient treatment alternative for VC in chronic hemodialysis.

## Material and Methods

### Hemodialysis condition

All (n = 45) were conventional hemodialysis with high flux of 4 hours, purely diffusive without hemodiafiltration, with a helixone dialyzer (Fresenius; CUF of 59 ml/h/mmHg, 1.8 m^2^, without variation throughout the study duration). The bath composition was: 1.25 mmol/L calcium, 35 mmol/L bicarbonate, 1,25 mmol/L potassium, 0,5 mmol/L magnesium and 140 mmol/L sodium.

### Samples and analytical parameters

45 patients in hemodialysis (form 46 to 80 years old, 26% women and 74% men) were studied. Pre- and post-hemodialysis blood samples were collected in heparin-containing tubes and immediately centrifuged at 4°C for 5 min at 5000 rpm. Plasma samples were frozen in liquid nitrogen and stored at -80°C until further use. For phosphate and ionized calcium quantification, the Phosphate Assay Kit (DIPI-500, BioAssay Systems, Hayward, CA) and Calcium Assays Kit (DICA-500, BioAssay Systems) were used, respectively. For quantification of phosphorus, total calcium, urea and p*H*, standard clinical analyses were used. This study was conducted according to the Declaration of Helsinki and approved by the Ethics Committee of Research of Universitary Hospital Fundación Jiménez Díaz. Participants were identified by a number and no other identifying material.

### Chemicals and inhibitors

All chemicals and inhibitors were obtained from Sigma-Aldrich, St Louis, MO.

### ALP assays

**Fig 1:**
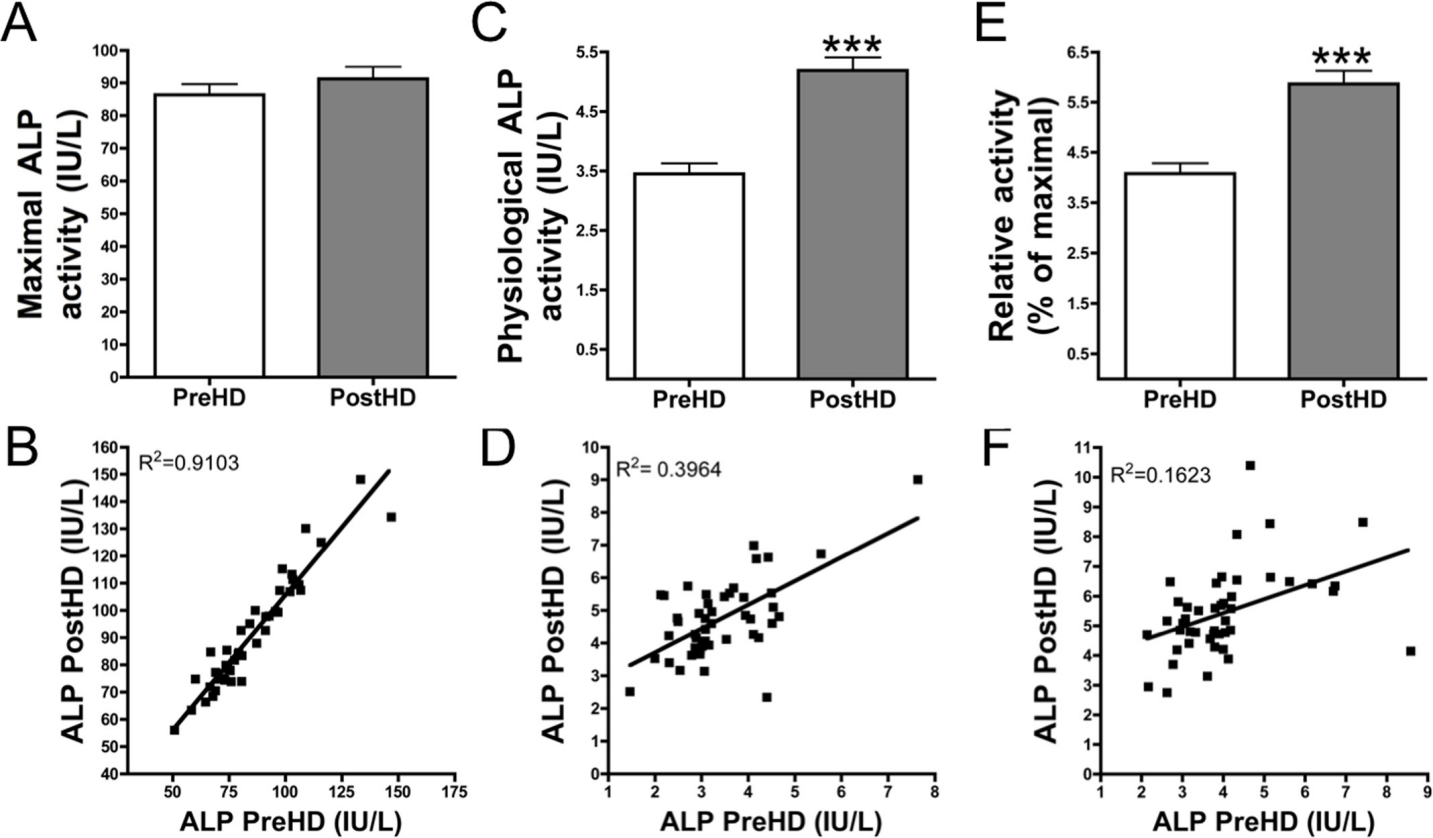
Physiological ALP activity increases post-hemodialysis. Maximal (**A**) and physiological (**C**) alkaline phosphatase (ALP) activities of pre-hemodialysis (preHD) and post-hemodialysis (postHD) plasma samples were quantified. For relative ALP activity (**E**), physiological ALP activities were divided by maximal ALP activities for each sample. (**A, C, E**) The Wilcoxon matched pairs test was used for statistical analysis. (**B, D, F**) The scattergraph demonstrated a correlation between pre- and post-hemodialysis ALP activities in maximal (**B**) and physiological (**D**) conditions, and relative ALP activity (**F**). Lineal regression demonstrated a significant deviation in all cases. *** *P* < 0.001.

**Fig 2:**
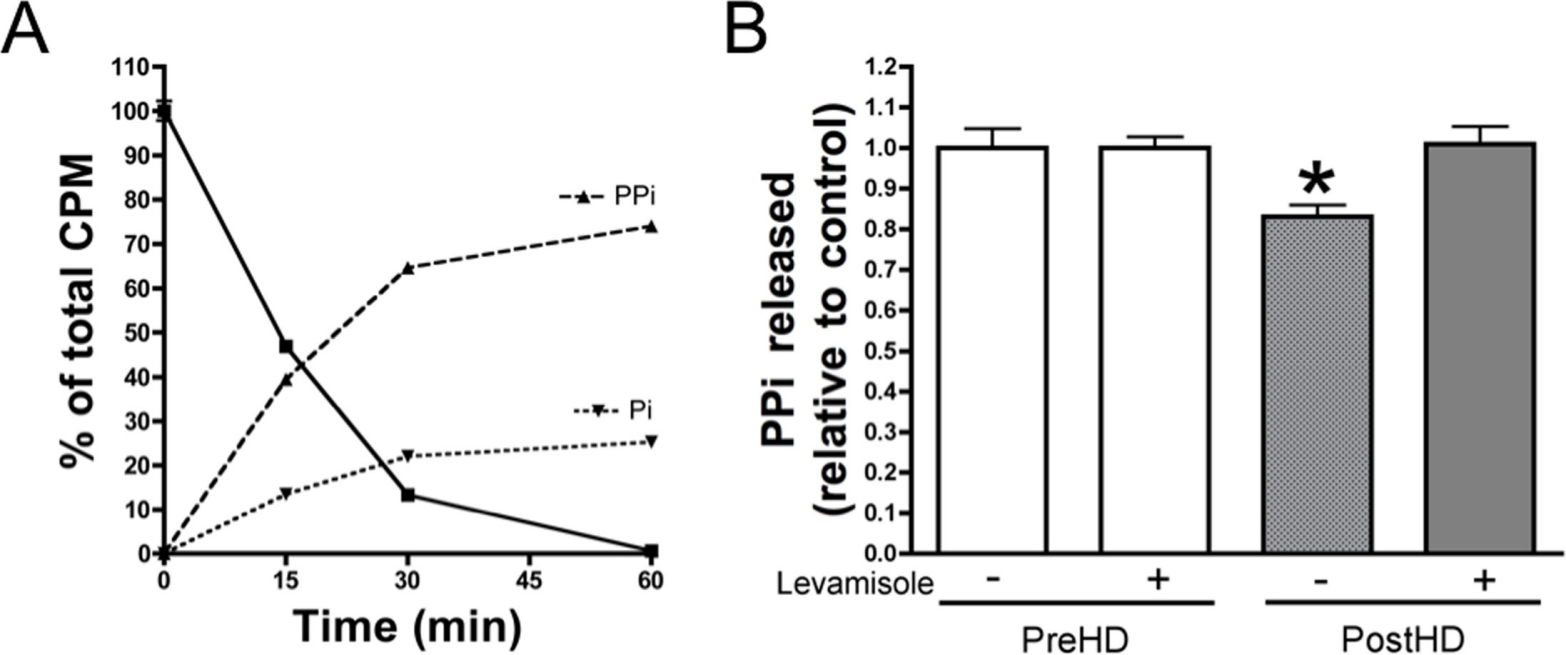
PPi synthesis remains unaltered post-hemodialysis. Thin layer chromatography. (**A**) Plasma hydrolysis of 1 μmol/L ATP showed PPi and Pi production at the indicated times. (**B**) PPi quantification at the indicated conditions and sample types following 1 hr of incubation with 1 μmol/L ATP and [γ^32^P]ATP as a radiotracer. Pre-hemodialysis plasmas without levamisole were used as controls. Results are represented as mean ± SEM for all plasma samples (n = 45). There was a significant difference in post-hemodialysis (postHD) without interaction (two-way ANOVA; *P* = 0.017). The Bonferroni post-test was used for statistical analysis. PreHD, pre-hemodialysis. PPi, pyrophosphate; Pi, phosphate. **CPM**: counts per minute. * *P* < 0.05.

ALP activity was measured using the Quantichrom Alkaline Phosphatase Assay Kit (DALP, BioAssay Systems) according to the manufacturer’s instructions or using modifications to its protocol. In all experiments, 50 μL of human plasma was incubated with 150 μL of the corresponding buffers. For maximal ALP activity (Fig 1A), plasma samples from patients were incubated with the working solution supplemented with p-Nitrophenyl phosphate (pNPP) at a final concentration of 10 mmol/L. For physiological ALP activity (Fig 1C), human plasma was incubated with dH_2_0 containing 10 mmol/L pNPP. For p*H* curve dependence (Fig 2A), plasma samples were incubated in dH_2_0 containing pNPP and Tris-HCl at a final concentration of 50 mM at the p*H* indicated. Samples were incubated for 2 hr and measured at 405 nm every 30 min. For ALP activity and p*H* curve dependence, samples were incubated for 30 min and measured every 5 min. Slopes and activities were calculated using linear regression (with GraphPad Prism 5).

### ATP hydrolysis assays

To analyze the products released during ATP hydrolysis, 50 μL of human plasma was incubated with 50 μL of Hank’s salt solution containing ATP and [γ^32^P]ATP at a final concentration of 1 μmol/L and 10 μCi/mL, respectively. Production of PPi, Pi, and ATP was determined by chromatography on PEI-cellulose plates, and they were developed with 650 mmol/L KH_2_PO_4_ p*H* 3 as previously described[8,18]. After radiography, spots were excised and counted by liquid scintillation.

### PPi quantification

**Fig 3:**
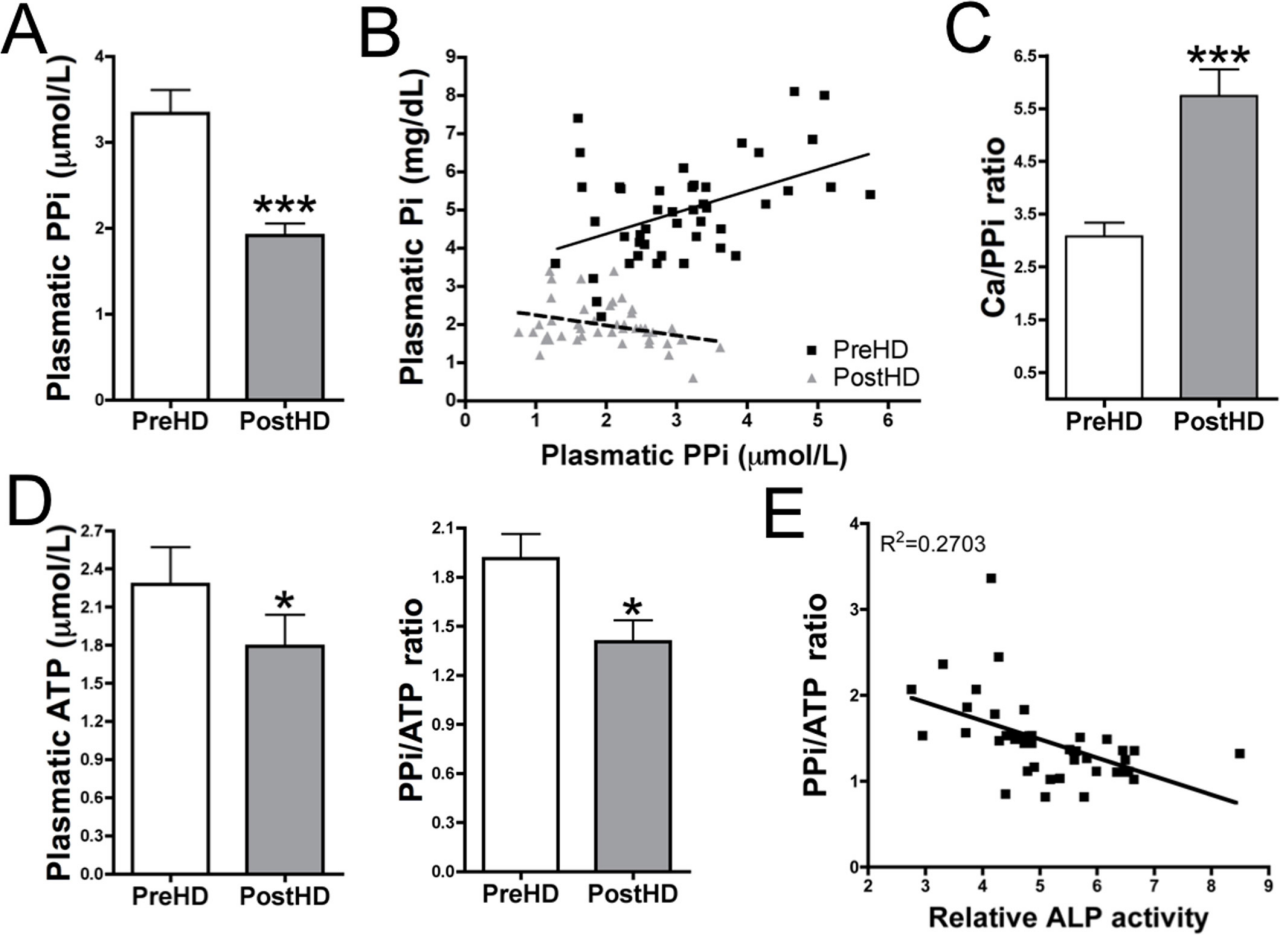
PPi availability is reduced post-hemodialysis. (**A**) Plasma PPi quantification. (**B**) The scattergraph shows a correlation between plasma Pi and PPi. (**C**) Plasma (Ca)/PPi ratio. (**D**) Plasma ATP quantification and PPi/ATP ratio. (**E**) The scattergraph shows a correlation between PPi/ATP ratio and relative ALP activity in PostHD plasma. (**B, E**) A significant deviation was observed using linear regression. (**A, C, D**) Results are represented as mean ± SEM for all paired samples (n = 45). Wilcoxon’s matched pairs test was used for statistical analysis. PPi, pyrophosphate; Pi, phosphate; Ca, calcium; ALP, alkaline phosphatase; PreHD, pre-hemodialysis; PostHD, post-hemodialysis. * *P* < 0.05; *** *P* < 0.001.

PPi was measured (Fig 3A) with an enzyme-linked bioluminescence assay as previously described[8].

### Statistical analyses

**Fig 4:**
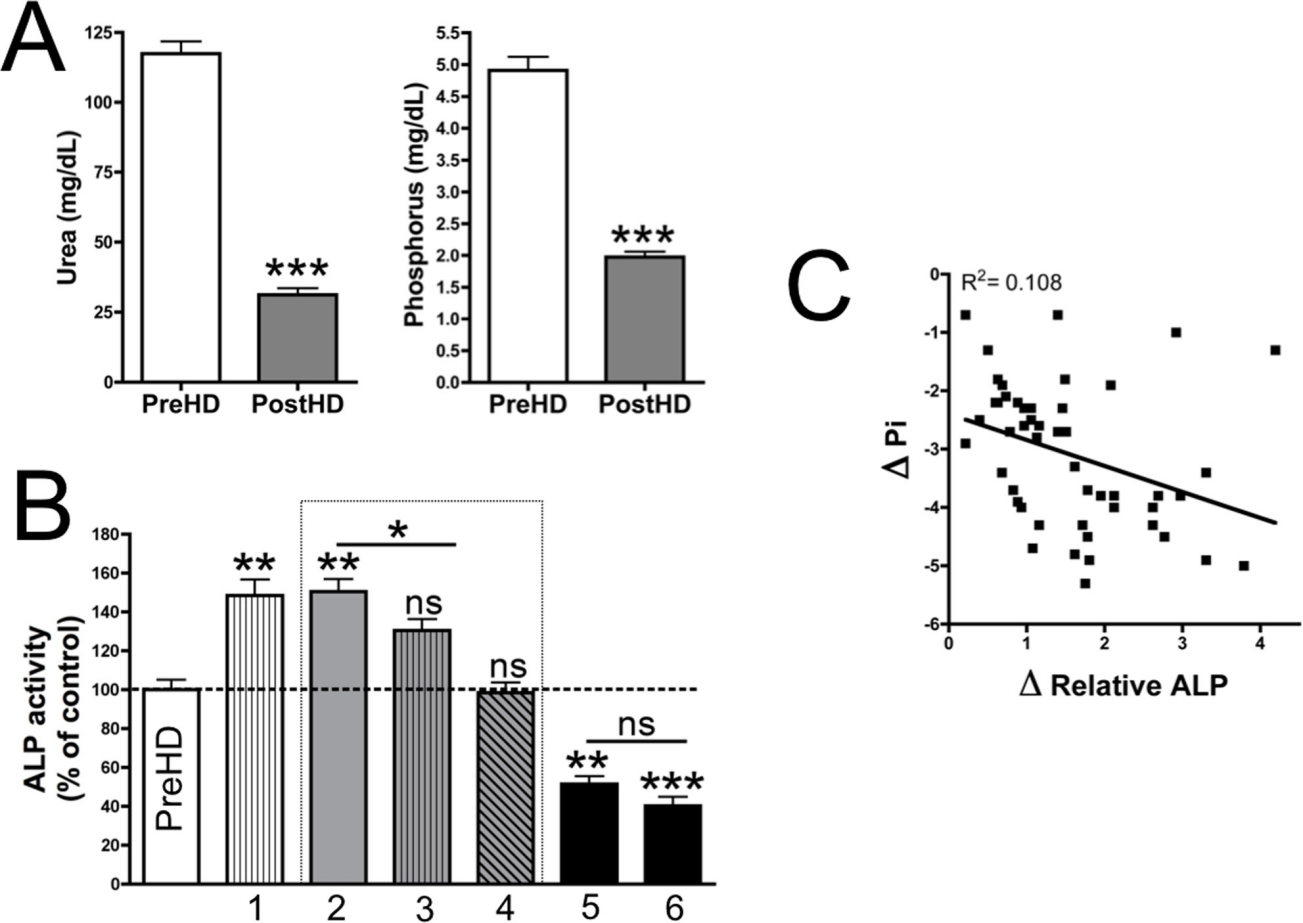
Plasma phosphate levels inhibit ALP activity. (**A**) PreHD (left) and postHD (right) levels of urea and phosphate in plasma. Results are represented as mean ± SEM (n = 45). Wilcoxon’s matched pairs test was used for statistical analysis. (**B**) ALP activities of post-hemodialysis samples (with the exception of the controls, which were pre-hemodialysis samples) without or with potential inhibitors: (**1**) 120 mg/mL (20 mmol/L) urea; (**2**) no addition; (**3**) 4.5 mg/dL (1.5 mmol/L) inorganic phosphate; (**4**) 9 mg/dL inorganic phosphate; (**5**) 20 mmol/L EDTA; and (**6**) 100 μmol/L levamisole. Results are represented as mean ± SEM (n = 45). For statistical analysis, Friedman’s one-way ANOVA test and Dunn’s post-test were used. (**C**) The scattergraph demonstrates a correlation between ΔPi (the difference in post- and pre-hemodialysis phosphate levels) and ΔALP (the difference in post- and pre-hemodialysis ALP levels). PreHD, pre-hemodialysis; PostHD, post-hemodialysis. * *P* < 0.05; ** *P* < 0.01; *** *P* < 0.001.

**Fig 5:**
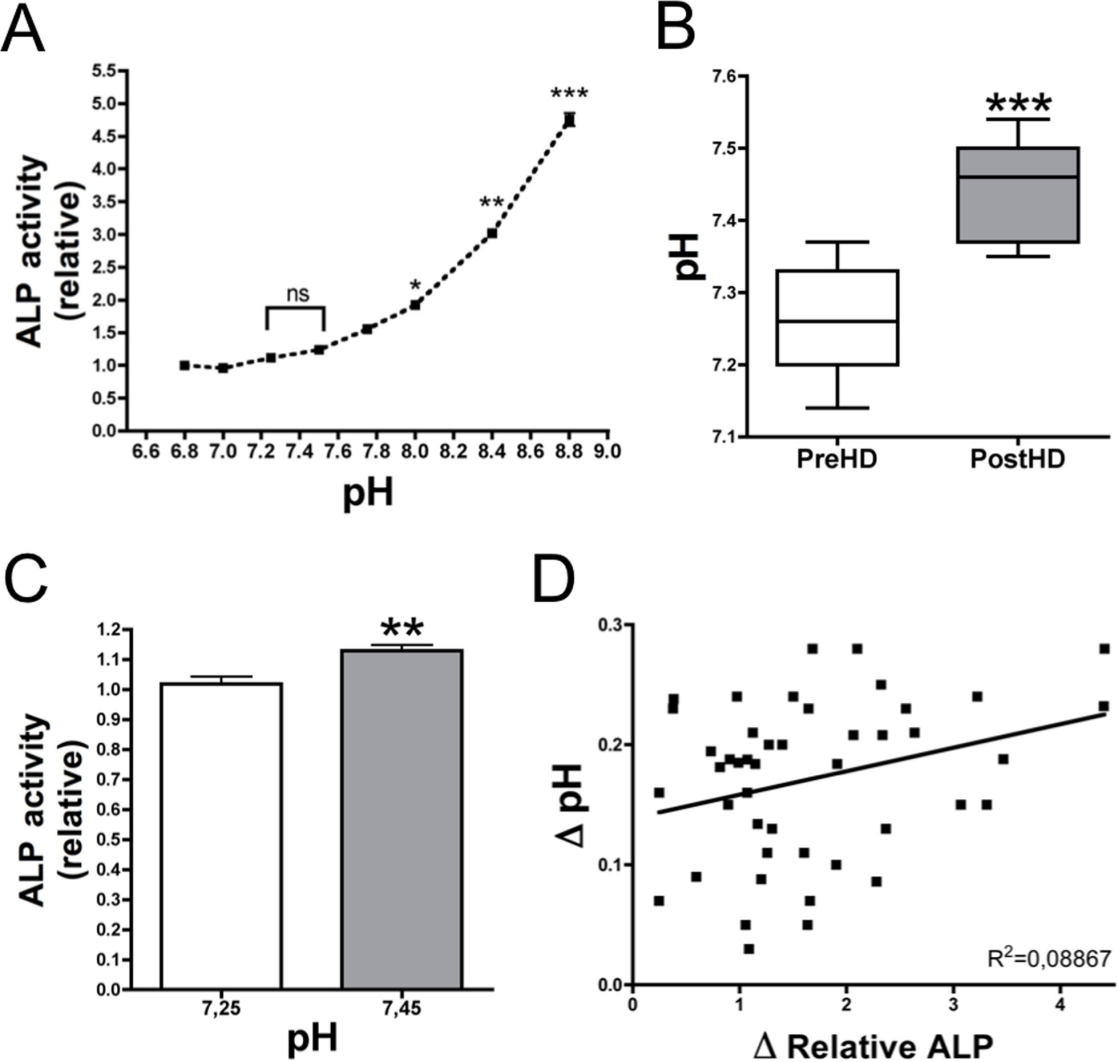
Influence of plasma p*H* on alkaline phosphatase activity. (**A**) A p*H*-dependent curve using ten pools comprising four different post-hemodialysis plasma samples per pool at the p*H* indicated. Friedman’s one-way ANOVA test and Dunn’s post-test were used for statistical analysis. (**B**) Box and whiskers graph demonstrating pre- and post-hemodialysis plasma p*H* values (preHD and postHD, respectively; n = 10). (**C**) ALP activity in post-hemodialysis plasmas was quantified at the p*H* indicated. (**B, C**) Plasma samples were collected from all patients (n = 45), and results were analyzed using the Wilcoxon matched pairs test. (**D**) The scattergraph demonstrates a correlation between Δp*H* (the difference in post- and pre-hemodialysis p*H*) and Δ Relative ALP (the difference in post- and pre-hemodialysis relative ALP levels). * *P* < 0.05; ** *P* < 0.01; *** *P* < 0.001.

Results are represented as mean ± standard error of the mean (SEM). Experiments shown in Figs 1–5 were performed using all pairs of samples (n = 45), with the exception of Fig 5A in which ten pools with four plasma samples per pool were used. Statistical significance was determined with GraphPad Prism 5. In Figs 1A, 1C, 1E, 3A, 3C, 3D, 4A, 5B and 5C the Wilcoxon matched pairs test was used for statistical analysis. In Fig 2B, two-way ANOVA and Bonferroni post-test were used for statistical analysis. In Fig 4B Friedman’s one-way ANOVA test and Dunn’s post-test were used for statistical analysis.

## Results

### ALP activity increases after hemodialysis in physiological conditions

Standard protocols for the quantification of ALP activity utilize a buffer that results in maximal ALP activity. The ideal formulation of this buffer induces maximal efficiency in ALP activity and has an alkaline p*H*. Using this method, the maximum activity of ALP, which strictly correlates with the amount of ALP enzyme present in serum or plasma, can be detected; however, ALP activity in physiological conditions, e.g., in normal plasmatic conditions, has not yet been studied. Therefore, we examined ALP activity in both maximal and physiological conditions. Maximal ALP activity was observed to slightly increase after the hemodialysis session (before hemodialysis, 87.4 ± 3.3 IU/L; after hemodialysis, 89.3 ± 3.6 IU/L), although all ALP activity values were found to be within the normal range (45–129 IU/L). Physiological ALP activity significantly increased (*P* < 0.001) in post-hemodialysis plasma (5.2 ± 0.2 IU/L) compared with pre-hemodialysis plasma (3.5 ± 0.2 IU/L) in all samples. Pre- and post-hemodialysis physiological ALP activities were found to be 4.1% and 5.9% of the maximal ALP activity, respectively (relative ALP activity). ALP activity in physiological conditions was observed to increase by approximately 40% after a hemodialysis session. There was a significant correlation between pre- and post-hemodialysis ALP activities in maximal (*P* < 0.001), physiological (*P* < 0.001), and relative (*P* = 0.011) ALP activities; however, the R^2^ values differed and poor in the case of physiological and relative ALP activities (Fig 1).

### PPi synthesis is not affected post-hemodialysis

As PPi hydrolysis increases via ALP in post-hemodialysis plasma, we wanted to determine whether PPi synthesis via ATP hydrolysis is altered. PPi production via hydrolysis of 1 μmol/L ATP was significantly decreased to 83.1% of pre-dialysis plasma in post-hemodialysis plasma (Fig 2). Addition of an ALP inhibitor (levamisole) resulted in recovery of impaired PPi synthesis, and a difference in PPi production by ATP hydrolysis was not observed between pre- and post-hemodialysis plasma. This result indicates that the reduction in PPi production is a consequence of the increase in ALP activity in post-hemodialysis plasma.

### Hemodialysis sessions reduce PPi levels

Plasma PPi levels were significantly decreased (42%; *P* < 0.001) post-hemodialysis (3.3 ± 0.3 μmol/L *vs*. 1.9 ± 0.1 μmol/L; Fig 3A). Although pre-hemodialysis PPi levels were found to range between 1.4 and 6.3 μmol/L, a decrease in PPi levels post-hemodialysis was observed in all paired samples analyzed. Moreover, a correlation with phosphate levels was found in both pre- and post-hemodialysis plasma (Fig 3B). A positive correlation was found between PPi levels in pre-hemodialysis and phosphate (*P* = 0.0014; R^2^ = 0.3125). This correlation was observed to be inverted in post-hemodialysis samples (*P* = 0.0292; R^2^ = 0.0213) due to the dramatic reduction in phosphate levels. Furthermore, both calcium and ionized calcium levels were significantly (*P* < 0.001) increased in post-dialysis plasma in 86.6% of paired samples analyzed (8.7 ± 0.1 mg/dL *vs*. 9.6 ± 0.1 mg/dL for calcium; and 6,56 ± 0.8 mg/dL *vs*. 8.4 ± 0.4 mg/dL for ionized calcium). The Ca/PPi ratio was also significantly (*P* < 0.001) increased, by approximately 2-fold (Fig 3C).

Plasma ATP levels were significantly decreased (21%; *P* = 0.03) post-hemodialysis (2.3 ± 0.3 μmol/L *vs*. 1.8 ± 0.3 μmol/L) but to a lesser extent than PPi (Fig 3D). The PPi/ATP ratio was significantly reduced (~26%; *P* = 0.02) post-hemodialysis (1.9 ± 0.2 *vs*. 1.4 ± 0.1). Furthermore, there was a significant correlation (Fig 3E) between the PPi/ATP ratio and relative ALP activity (*P* = 0.003), suggesting that the reduction in the PPi/ATP ratio is a consequence of the increase in ALP activity.

### Phosphate inhibits ALP activity

Urea and phosphate are two known uremic toxins that are removed during hemodialysis. Urea and phosphate levels were significantly reduced post-hemodialysis (3.8-fold and 2.5-fold, respectively) (Fig 4A). Addition of phosphate in post-hemodialysis plasmas significantly decreased ALP activity, although addition of urea did not elicit a similar effect (Fig 4B). EDTA and levamisole were used as controls of ALP inhibition. There was significant deviation between decreased phosphate concentrations and the increase in relative ALP activity post-hemodialysis (Fig 4C). This correlation was not found for maximal ALP activity.

### Role of p*H* in ALP activity in hemodialysis patients

As shown Fig 5A, ALP activity was observed to increase at an alkaline p*H*. Post-hemodialysis plasma p*H* (7.45 ± 0.02) was significantly increased (*P* < 0.001) compared with the pre-dialysis p*H* (7.26 ± 0.02) in all samples analyzed (Fig 5B). The slight variation in p*H* (approximately 0.2 units) was observed to induce a significant increase in ALP activity (approximately 9%) at a p*H* of 7.45 compared with a p*H* of 7.25 (Fig 5C). Finally, a significant correlation was found between the variation in p*H* (Fig 5D) and the variation in the Relative ALP activity (*P* = 0.0470; R^2^ = 0.08867).

## Discussion

More than 5000 substances accumulate as a consequence of renal dysfunction, and produce complex and multiple biochemical and physiological abnormalities (uremic syndrome). Phosphate (Pi) and hydrogen ions (H^+^) are the most common substances that accumulate in blood as a consequence of renal dysfunction, resulting in hyperphosphatemia and acidosis in chronic hemodialysis patients[2,19].

In our study, extraction of uremic toxins was demonstrated to dramatically increase ALP activity in physiological conditions (Fig 1). ALP is involved in PPi degradation. Inhibition of ALP did not alter PPi synthesis from ATP hydrolysis (Fig 2). PPi plays an important role in the development of VC due to its capacity to prevent CPC deposition. Plasma PPi levels was found to be reduced by 42% post-hemodialysis (Fig 3C). Despite the loss of a small amount of ATP (Fig 3D) during hemodialysis (the source of PPi), the PPi/ATP ratio was significantly reduced post-hemodialysis (Fig 3D). Furthermore, the increase in ALP activity was significantly correlated with the decrease in phosphate levels post-hemodialysis (Fig 4C). These data support our findings on ALP activity.

Elevated levels of phosphate are a risk factor for VC, cardiovascular disease, and mortality[2]. In chronic hemodialysis patients, phosphate is considered a uremic toxin that requires elimination. To reduce plasma phosphate levels, phosphate binders and dietary phosphate restriction are currently used to support dialytic phosphate clearance. Elimination of plasma phosphate levels reduces calcium phosphate products; however, phosphate clearance was found to increase ALP activity under physiological conditions (Fig 4). A decrease in phosphate levels correlated with increases in ALP activity post-hemodialysis (Fig 4C). On the other hand, less than 1/5 of this increase may be explained by post-hemodialysis-associated alkalosis (Fig 5). Alkalosis plays an important role in VC as a high p*H* increases the formation of CPC due to a rightward shift in the H_2_PO_4_^-^/HPO_4_^2-^ equilibrium[20]. In the present study, p*H* values were observed to increase by 0.2 post-hemodialysis. This slight increase resulted in a significant increase in ALP activity (Fig 5C). Therefore, alkalinization-associated hemodialysis may affect the amount of CPC deposited in soft tissues due to an increase in both CPC synthesis and PPi hydrolysis.

Calcium-phosphate product (CaxPi) is associated with an increased risk of VC[3,4]. Several studies demonstrated that calcium levels play a key role in CPC formation[20,21]. An increase in calcium levels and a decrease in phosphate levels, to maintain a constant CaxPi, induce CPC formation[20]. Interestingly, CPC formation is not observed when phosphate levels are elevated in association with reduced calcium levels[21]. During hemodialysis clearance, phosphate and pyrophosphate levels are reduced, although calcium levels are elevated. These findings suggest that the procalcifying condition is elevated during dialysis due to alkalinization (Fig 5) and an increase in the Ca/PPi ratio (Fig 3C) and ALP activity (Fig 1).

Plasma PPi concentration was reported to be reduced by 32% after standard hemodialysis, primarily as a consequence of dialytic clearance[22]. The authors explained that PPi deficiency does not appear to be explained by altered mineral metabolism in chronic hemodialysis patients. The aforementioned study found a correlation between pyrophosphate and Pi, which was previously reported[23], but not with ALP or other plasma parameters. The authors analyzed the clinical or maximal ALP activity but not ALP activity under physiological conditions. In the present study, we also found a reduction in PPi levels post-hemodialysis (Fig 3A), which can be explained by loss of ATP/ PPi during the dialysis clearance and also by increments in the ALP activity associated with uremic toxin clearance and alkalosis (Fig 3D, reduction in PPi/ATP ratio).

These new findings may support a new therapeutic treatment against ALP during dialysis to reduce PPi hydrolysis. Recent studies using rat and mice models showed that daily injections of exogenous PPi prevent VC[8,24–26]. Given that hemodialysis session is a perfect storm for VC hardly unavoidable, administration of exogenous PPi during hemodialysis is a potential approach for the prevention of VC. Addition of exogenous PPi alone will be dramatically hydrolyzed by increased ALP activity; therefore, PPi should be administered in combination with ALP inhibitors to increase its availability. These data can help us in the design of a more effective clinical trial for these patients.

In conclusion, our study revealed three important concepts. First, measurements of ALP in clinical practice represent the maximal activity in ideal conditions but not the actual activity under normal or physiological conditions. Physiological ALP activities represent 4–6% of the maximal activity. Second, physiological ALP activity increases after dialysis clearance, reducing PPi availability. Finally, the increase in ALP activity may be primarily explained by a decrease in phosphate and H^+^ levels during hemodialysis.
